# A Cluster-Randomised Trial Evaluating an Intervention for Patients with Stress-Related Mental Disorders and Sick Leave in Primary Care

**DOI:** 10.1371/journal.pctr.0020026

**Published:** 2007-06-01

**Authors:** Ingrid M Bakker, Berend Terluin, Harm W. J van Marwijk, Daniëlle A. W. M van der Windt, Frank Rijmen, Willem van Mechelen, Wim A. B Stalman

**Affiliations:** 1 Institute for Research in Extramural Medicine, VU University Medical Center Amsterdam, Amsterdam, The Netherlands; 2 Department of General Practice, VU University Medical Center Amsterdam, Amsterdam, The Netherlands; 3 Department of Clinical Epidemiology and Biostatistics, VU University Medical Center Amsterdam, Amsterdam, The Netherlands; 4 Department of Public and Occupational Health, VU University Medical Center Amsterdam, Amsterdam, The Netherlands

## Abstract

**Objective:**

Mental health problems often affect functioning to such an extent that they result in sick leave. The worldwide reported prevalence of mental health problems in the working population is 10%–18%. In developed countries, mental health problems are one of the main grounds for receiving disability benefits. In up to 90% of cases the cause is stress-related, and health-care utilisation is mainly restricted to primary care. The aim of this study was to assess the effectiveness of our Minimal Intervention for Stress-related mental disorders with Sick leave (MISS) in primary care, which is intended to reduce sick leave and prevent chronicity of symptoms.

**Design:**

Cluster-randomised controlled educational trial.

**Setting:**

Primary health-care practices in the Amsterdam area, The Netherlands.

**Participants:**

A total of 433 patients (MISS *n* = 227, usual care [UC] *n* = 206) with sick leave and self-reported elevated level of distress.

**Interventions:**

Forty-six primary care physicians were randomised to either receive training in the MISS or to provide UC. Eligible patients were screened by mail.

**Outcome Measures:**

The primary outcome measure was duration of sick leave until lasting full return to work. The secondary outcomes were levels of self-reported distress, depression, anxiety, and somatisation.

**Results:**

No superior effect of the MISS was found on duration of sick leave (hazard ratio 1.06, 95% confidence interval 0.87–1.29) nor on severity of self-reported symptoms.

**Conclusions:**

We found no evidence that the MISS is more effective than UC in our study sample of distressed patients. Continuing research should focus on the potential beneficial effects of the MISS; we need to investigate which elements of the intervention might be useful and which elements should be adjusted to make the MISS effective.

## Editorial Commentary

**Background:** People who take sick leave from work as a result of mental health problems very often report that the cause is stress-related. Although stress-related sick leave imposes a significant burden on individuals and economies, few evidence-based therapies exist to prevent sick leave in people who are experiencing stress-related mental health problems. The researchers carrying out this study wanted to evaluate the effectiveness of an intervention for stress-related mental health disorders amongst people who had been on sick leave for less than three months. The intervention involved short training sessions for primary health-care practitioners, during which the practitioners were taught how to diagnose stress-related problems; how to provide information to patients and encourage their recovery and active return to work; and how to give advice and monitor patients' recovery. The researchers carried out a cluster-randomized trial evaluating this training program, in which 46 primary care practitioners were assigned by chance to receive either the training program or to practice usual care. Over the course of the trial, 433 patients with elevated levels of distress and sick leave were included in the study, 227 of whom were treated by practitioners receiving the training program and 206 of whom received usual care. These patients were followed up for 12 months and the primary outcome studied in the trial was the length of sick leave taken until full return to work. Secondary outcome measures included patients' reports of distress, depression, and other symptoms as recorded using specific questionnaires.

**What the trial shows:** In the trial, data on the primary outcome measure was available for 87% of the patients treated by practitioners receiving the training intervention and 84% of the patients receiving usual care. When these outcomes were analyzed, there was no evidence of a benefit of the training program on amount of sick leave taken. Over the course of the study, the severity of patients' self-reported symptoms fell in both groups, but there was no significant difference in symptom severity between the two groups of patients. A subgroup analysis suggested that more practitioners in the intervention group recognized patients as having stress-related mental health problems. Among the group of patients who were diagnosed as having stress-related mental health problems, those who were treated by practitioners in the intervention group seemed to return to work slightly more quickly than those in the usual care group. However, it is not easy to interpret the findings of this secondary analysis.

**Strengths and limitations:** Strengths of this study include the procedures for cluster randomization, in which primary care practitioners were randomized, rather than patients. This process ensures that only patients assigned to the intervention arm receive the benefits of the intervention, and avoids “contamination” between intervention and control groups. A further strength includes the blinding of researchers who were collecting data to the intervention that each practitioner had received. The findings of the study, however, are difficult to interpret. No effect of the training intervention was found on the study's primary outcome measure; it is possible that the training intervention does indeed have some benefit, but the benefit may not have been found in this particular trial because of the inclusion of patients with a very wide range of problems; in addition the practitioners may have not had the time or ability to apply what they learnt in the training program.

**Contribution to the evidence:** Very little evidence exists regarding the effects of training interventions for improving care of patients with stress-related mental health problems. The findings of this trial support those of another study carried out in a primary care setting, which found that training interventions do not seem to reduce length of sick leave. However, another study carried out in an occupational health-care setting, in which patients included in the trial had been recognised as having stress-related mental disorders, did find some benefit of an intervention program.

## INTRODUCTION

Mental health problems often affect functioning to such an extent that they result in sick leave [1]. The worldwide reported prevalence in the working population is 10%–18% [2,3]. These problems cause a public health burden resulting in enormous personal and financial costs [4–7]. Sick leave often lasts for a long period of time, and in developed countries, mental health problems are one of the main grounds for receiving disability benefits [8,9]. In up to 90% of mental health problems the cause is stress-related [4–6,10] and health-care utilisation is mainly restricted to primary care [9].

Common psychopathology, as seen in primary care, often starts with failure to cope with personal, social, or occupational demands. The ability to cope or readjust is overtaxed, and this increases the probability that psychological distress will follow [11–13]. Sick leave indicates a process of depleting psychological resources; the patient has stopped trying to cope, and gives in. When not due to more severe psychiatric conditions such as depressive disorder or anxiety disorder, this condition is known as adjustment disorder (Diagnostic and Statistical Manual of Mental Disorders, fourth edition) [14,15], neurasthenia (International Classification of Diseases, tenth revision) [16], or nervous breakdown. Because such patients are labelled with a number of inter-related terms and definitions, we use the term stress-related mental disorder (SMD) to indicate relevant dimensions of psychopathology that are subacute, but not yet chronic, and clearly related to stress. Subsequently to SMDs, persistent distress contributes to more severe psychopathology and chronic conditions such as depression and anxiety disorders [17].

As yet, there are no evidence-based primary care interventions to improve functioning and to prevent long-term sick leave in patients with SMDs. Primary care physicians (PCPs) are not always aware of the potentially harmful consequences of sick leave and stress, because the symptoms seem to be self-limiting. Or, PCPs may be overly cautious and not question the continuation of sick leave nor ask the patient to make more effort to cope with the situation, feeling that this type of response undermines the mutual trust between patient and doctor [18,19].

## METHODS

The present study is a cluster-randomised controlled effectiveness trial in which PCPs were randomised to an intervention group that was trained to deliver a minimal intervention for stress-related mental disorders, or to a control group that delivered care as usual. Distressed patients on sick leave visiting the practices of both PCP groups were screened, included, and followed up for one year [20]. The Medical Ethics Committee of the VU University Medical Center approved the study protocol and procedures.

### Participants

We approached 139 PCPs in two districts where the Department of General Practice of the VU University Medical Center has some type of network positioned. A total of 46 PCPs signed informed consent, both for participating in our trial and for being randomised to either the intervention training or to the usual care (UC) group.

**Figure 1:**
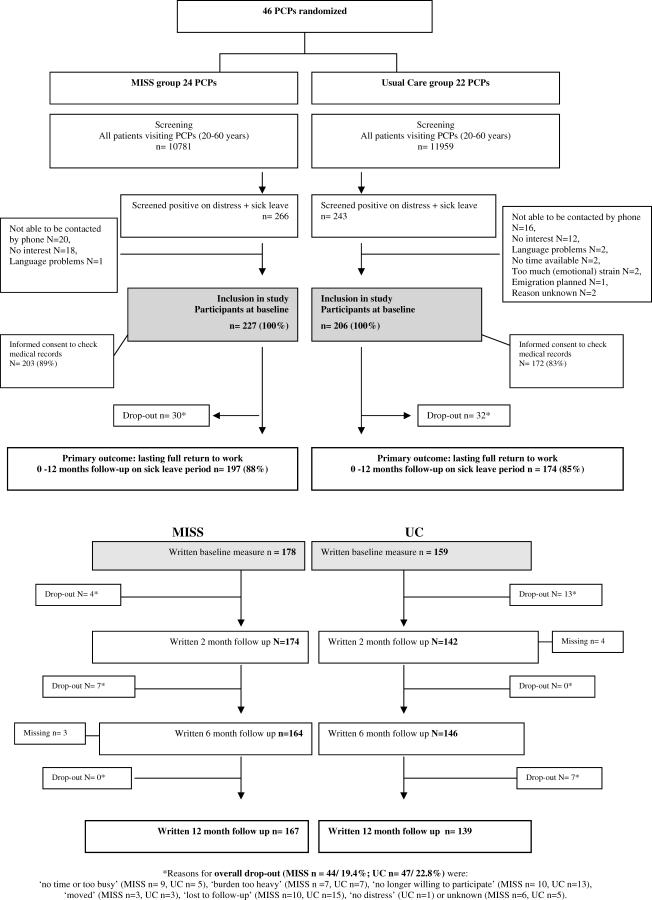
CONSORT Flowchart

**Figure 2:**
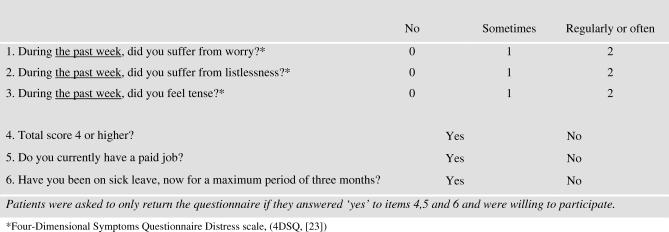
Patient Screener

In order to recruit enough eligible patients, we made use of the computerised patient record system and approached the source population of patients (*n* = 22,740, see Figure 1) by mail. The source population consisted of all primary care attenders (20–60 y) who visited consulting hours of the participating PCPs. PCPs excluded only patients with very severe psychiatric disorders (mania or psychosis), patients with terminal illness, or patients with an inadequate command of the Dutch language. The source population of attenders was asked to respond only if they met our criteria. Patient inclusion criteria were symptoms of SMD, and sick leave for no longer than three months from a paid job. Symptoms of SMD were measured by means of self-reported levels of distress (e.g., worrying, listlessness, feeling tense—see Figure 2) in order to recruit patients. We approached the source population every one or two weeks, until a sufficient number of patients from a particular PCP were enrolled. Final recruitment took place by phone survey. All patients who had returned the questionnaire and screened positive on distress and sick leave were contacted. Next, the inclusion criteria on distress and sick leave for no longer than three months were checked again. Since there are no diagnostic criteria, we did not attempt to make a diagnosis of SMD. If positive, patients were asked for their informed consent to be included in the study and to have their data collected and analysed. If the patient consented, the telephonic baseline interview was started. This method of recruitment, unaffected by the PCPs' diagnostic or therapeutic behaviour, ensured that the recruited patients included in the intervention and control groups were comparable, and at least not subjected to selection bias.

### Interventions

Over a period of 6–10 wk, the PCPs randomised into the intervention group received training in the Minimal Intervention for Stress-related mental disorders with Sick leave (MISS). The training comprised two sessions of 3.5 h and two regular follow-up sessions of 2 h (total 11 h). The tutors during the training were the PCP who developed the intervention (BT) and an occupational physician. During the training, the PCPs were instructed to use specific methods of communication to help the patient, within three consultations on a time-contingent course, to achieve functional recovery. The MISS takes into account the time constraints under which a PCP works, as well as the position of a PCP as a generalist who does not have the capacity to apply highly specialised interventions. The necessary set of skills was clearly defined and taught to the PCPs. First, the PCPs were taught to *diagnose an SMD,* and to detect symptoms of depression and anxiety. They were then taught how to give *information* and promote the patient's understanding and how to emphasise the importance of the patient's active role with regard to successful return to work. Subsequently, they practised giving *advice* on the content of functional rehabilitation. Furthermore, the PCPs were taught active *monitoring* to evaluate whether the patient had made efforts to translate the (work) situation into a problem that could be solved. Lastly, the PCPs were instructed to consider *referral* to more specialised care in case no progress had been made, since the patient was not likely to benefit from more time off work. The PCPs in the UC group received no information or advice about the content of the intervention beforehand, but were offered the training at the end of the trial. Guidelines for PCPs are available for the treatment of depression [21] and anxiety [22], but not yet specifically for SMDs.

Actual treatment of the participating patients was left to the discretion of the PCPs, who were informed of a patient's participation only after a month. At baseline, patients were asked whether or not they had planned another visit to their PCP. If not, they were asked if they were considering another visit. The PCP was not obliged to apply the MISS or any other intervention, nor were the patients obliged to visit their PCP.

### Objectives

The aim of this study was to assess the effectiveness of our MISS in primary care, which is intended to reduce sick leave and prevent chronicity of SMD symptoms in patients. We hypothesised that the MISS would be more effective than UC, particularly in patients who had been diagnosed with SMD by the PCP.

### Outcomes

The primary outcome was duration of sick leave in calendar days from the first day of sick leave until full (not part-time) return to work, lasting for a period of at least 4 wk without partial or full relapse into sick leave. Patients were asked to record their days of sick leave, and this information was collected at baseline and after 2, 6, and 12 mo during telephone interviews. The secondary outcome measures were self-reported symptoms of distress, depression, anxiety, and somatisation. These were measured with the Four-Dimensional Symptom Questionnaire (4DSQ [23]) at baseline and at 2, 6, and 12 mo by mailed questionnaires. Elevated depression, anxiety, and somatisation scores are indicative of the existence of a depressive, anxiety, or somatisation disorder, whereas, in the absence of elevated depression, anxiety, and somatisation scores, an elevated distress score is indicative of an SMD. Two months after the baseline assessment, the PCPs in both groups were asked to fill in a structured questionnaire on the care provided and any diagnoses or working hypotheses in the past 3 mo according to their electronic medical record. All outcomes were measured at individual level.

### Sample Size

To estimate the required sample size, we used a method that takes into account potential clustering of effects within practices, the expected difference in outcome between intervention groups and the required power of the study. Sample size calculation was done with nQuery Advisor (Statistical Solutions, http://www.statsol.ie/html/nquery/nquery_home.html). A related study completed in occupational health care showed a difference of 15% in full return to work after a period of three months [24], which we considered to be a relevant difference for our trial. Expecting a proportion still on sick leave after 3 mo of 21% in the MISS group and 36% in the UC group, the sample size needed in each group was 126 (with a power of 80% at a 0.05 level two-sided log-rank test for equality of survival curves). Taking into account an intracluster correlation coefficient of 0.025 because of randomisation at physicians' level and seven patients per cluster, a total of 290 patients was needed. Assuming a dropout rate of 30% (approximately 10% at each moment of follow-up), enrolment of 415 patients was needed.

### Randomisation and Blinding

The PCPs were randomly allocated at four different recruitment moments, with block sizes of *n* = 10, *n* = 7, *n* = 14, and *n* = 15. A standard procedure was followed to conceal allocation: the names of the PCPs (and dummy in an uneven group) were put on a list in random order. Independently, a randomly ordered list of codes (1 = MISS, 2 = UC) was generated. These lists were brought together and the first PCP on the list was allocated to the group indicated by the first code, and so on. As a result, 24 PCPs were allocated to the MISS group and 22 to the UC group. After the PCPs were assigned and the MISS group had received 7 h of training, the patients were enrolled by screening the source population. Patient selection was performed by the research team, in order to prevent selection bias due to the MISS training. The PCPs entered the names and addresses of the source population, and the source population was given the inclusion criteria through a screening questionnaire. The research assistance team contacted the patients who returned the questionnaire by phone, gave information about the study, and was responsible for the final recruitment. The internal research team, responsible for the process of data collection, knew the study involved was a randomised controlled trial, but they had no information on which PCP was allocated to what condition. Patients and external interviewers were blinded. They were kept unaware that two different groups were formed, and were told that the study was about stress and sick leave.

### Statistical Methods

To evaluate the effectiveness of the MISS compared to UC, we used Cox regression analysis in STATA 8.0 (Stata, http://www.stata.com/stata8/) with robust standard errors [25] on our primary outcome measure. Differences in duration of sick leave were expressed as hazard ratios (HRs) and corresponding confidence intervals (95% confidence interval [CI]) for the MISS group, compared to the UC group. Estimates of the intervention effects on our secondary outcome measure were obtained from linear mixed models in SPSS 12.0 (SPSS, http://www.spss.com).

All analyses were conducted according to the intention-to-treat principle and corrected for the clustered design. The analyses were performed in several stages. First, baseline similarity of the two groups was examined for all potential confounders (age, gender, marital status, level of education) and baseline values of symptom scores (distress, depression, anxiety, and somatisation). Secondly, the unadjusted association between the groups (MISS versus UC) and both outcome measures were calculated. This association was then adjusted for each of the potential confounders separately. A forward selection procedure was followed to include the potential confounders. For our primary outcome measure this was done in order of highest change in the regression coefficient. Only those factors that changed the regression coefficient by more than 10% were considered to be confounders, and retained in the model. For our secondary outcome measure this was done by checking the significance of the *p*-values. Confounders were retained if they significantly contributed to the model (*p* < 0.05).

Furthermore, we were interested in potential modification of the treatment effects by the PCPs' diagnosis of SMD, and therefore preplanned subgroup analyses on diagnosis in the Cox regression analysis and linear mixed models effect evaluation. Baseline measures of self-reported symptoms, as well as diagnoses from the electronic medical records were examined, so we were able to check for classification of patients and severity of complaints (inclusion is only by self-reported level of distress with sick leave). We added product terms for the possible effect-modifier “diagnosis” (with categories SMD, other mental health problems, or somatic health problems) and condition (MISS or UC) to the model and checked for significance of the interaction term (*p* < 0.10). If significant, we proceeded with subgroup analyses. Since the diagnostic behaviour of the PCPs in the MISS group might have been changed as a result of the training, we were aware of confounding by selection bias. If the MISS PCPs detected more patients with an SMD than their UC counterparts, they would possibly detect a significantly higher proportion of patients with relatively mild disorders, which in itself could explain any differences in the patient outcomes of the groups. Therefore, we tested again for confounding of the association between the intervention and the outcome by baseline values of symptom scores (distress, depression, anxiety, and somatisation).

## RESULTS

### Participant Flow, Baseline Data, and Numbers Analysed

Between September 2003 and October 2004, a screening letter was sent to the source population of 22,740 patients. The overall response percentage on our screening method was 51.5%; this was measured in a group of 336 randomly selected attenders. A total of 433 patients (1.9% of 22,470) were included in the study, 66.3% of whom were women. Table 1 shows that baseline demographics and clinical characteristics of patients were largely similar, and only a small difference in level of education was found. The mean number of visits to the PCP, counted from the day of sick leave up to 3 mo, was 2.55 (standard deviation [SD] 2.12) in the MISS group, and 2.50 (SD 2.23) in the UC group (p = 0.839). With regard to clinical characteristics, baseline measures of self-reported symptoms were taken into account. Up to 80% of the patients scored above threshold on self-reported symptoms of distress, almost half scored above threshold on symptoms of depression, and about one-third scored above threshold on symptoms of anxiety. Symptoms of somatisation also were above threshold in more than half of the patients.

**Table 1:**
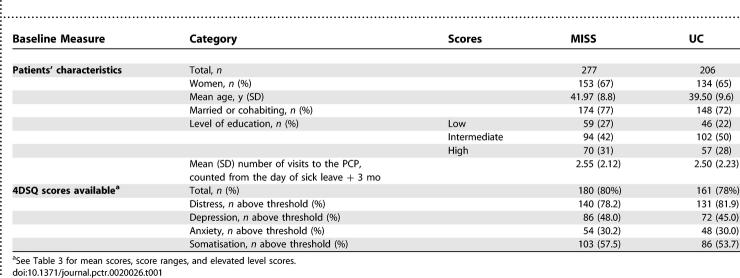
Characteristics of Patients with SMD Symptoms and Less Than Three Months Sick Leave

The participant flow from baseline up to 12 mo follow-up is represented in a diagram (Figure 1). For our primary outcome measure—duration of sick leave—data on 197 of the 227 (87%) of the patients from the MISS group and 174 of 206 (84%) of the patients from the UC group were available. During follow-up, 44 (19.4%) of the patients in the MISS group and 47 (22.8%) of the patients in the UC group withdrew from the study (see Figure 1). Only small differences were found with regard to baseline demographics and the clinical characteristics measured with the 4DSQ scores between the drop-outs and completers.

### Outcomes and Estimation

All analyses were adjusted for the clustering effect of PCPs. Tables 2 and 3 present the scores for primary and secondary outcome measures. Analysis showed no superior overall effect of the MISS on our primary outcome measure, days of sick leave (unadjusted HR 1.06, 95% CI 0.87–1.29; see Table 3). The median number of sick leave days before return to work was 96 (95% CI, 81–111) in the MISS group and 102 (95% CI, 75–182) in the UC group. Multilevel analyses showed that the analyses on our secondary outcome measure needed to be adjusted for the correlation of repeated measures within patients. Over 12 mo follow-up, the severity of all symptoms was reduced significantly in both groups (*p* < 0.001), and on our secondary outcome measure no significant differences were found between the MISS group and the UC group. A considerable number of patients still scored above threshold on self-reported symptoms after 12 mo follow-up. As can be seen in Table 3, this accounts for around 40% of the patients on symptoms of distress, and about one-quarter of the patients on symptoms of depression.

**Table 2:**
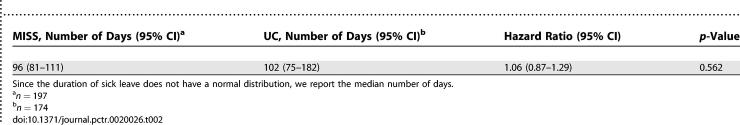
Median Number of Days of Sick Leave Before Lasting Full Return to Work

**Table 3:**
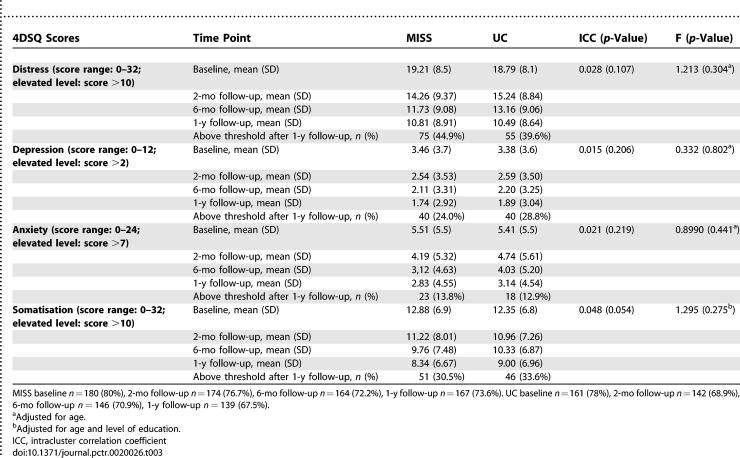
Symptom Scores at Follow-Up

### Ancillary Analyses

The baseline diagnoses from the medical records are shown in Table 4, divided into three categories: SMDs, other mental health problems, and somatic problems. As can be seen in Table 4, more PCPs in the MISS group recognised patients as having SMD (*p* = 0.068). These diagnosis categories showed interaction with the intervention in the Cox regression analysis on differences in duration of sick leave (*p* = 0.033). The PCPs' diagnosis of both SMDs and other mental health problems was associated with a longer median duration of sick leave, compared to the diagnosis of somatic health problems. Table 5 shows the subgroup analyses, and among patients diagnosed with SMDs, time to return to work was shorter in the MISS group than in the UC group (unadjusted HR 1.49 [0.98–2.26], and adjusted HR 1.72 [1.18–2.51]). The HRs for return to work in the subgroups other mental health problems and somatic problems slightly favoured the UC group. However, these differences were small and not statistically significant. For our secondary outcome measure, severity of symptoms, the interaction of intervention with diagnosis showed no significant results, so no subgroup analyses were performed. Although the subgroup analyses were planned out before the trial took place, in no case can this result be regarded as evidence for a difference between the MISS group and UC group.

**Table 4:**
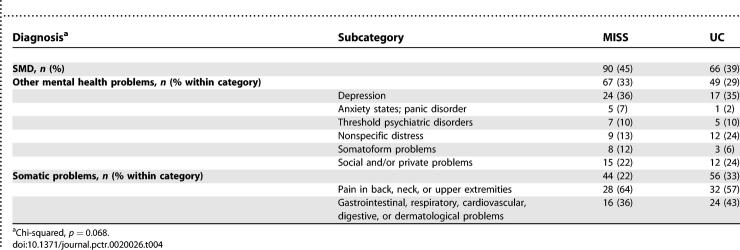
Diagnosis by Primary Care Physician at Baseline

**Table 5:**
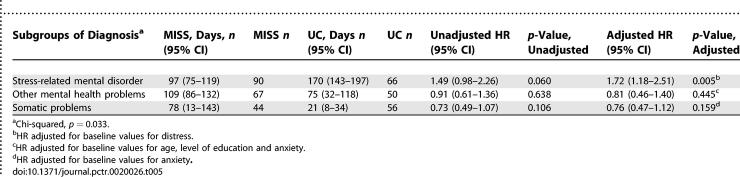
Median Number of Days of Sick Leave Before Lasting Full Return to Work, By Subgroups

## DISCUSSION

### Interpretation

We were unable to prove our hypothesis that the MISS would be more effective than UC, either on our primary outcome measure nor on the secondary. The median number of days on sick leave before return to work was substantial in both groups and a considerable number of patients still scored above threshold on the self-reported symptoms. However, the severity of symptoms was reduced significantly during the 1 y follow-up.

The MISS did not show an overall effect in our study sample of patients with symptoms of distress on sick leave. Possible explanations for the failure to find an effect can be sought within the patients, the PCPs, and the intervention used. First of all, we shall discuss the patients. There might have been a problem with the inclusion criteria used. It is possible that we misclassified a substantial part of the patients because our criteria might have been much too broad at both sides of the severity continuum. As can be seen with the self-reported symptoms of depression, 24.0% in the MISS group and 28.8% in the UC group still scored above threshold level after one year. This substantial group of patients may have had conditions that were of a more chronic nature (e.g., depressive disorder) and needed more extensive care. The difficulty here is that relevant dimensions of psychopathology that are subacute, but not yet chronic, and clearly related to stress (e.g., SMD), could not be distinguished in a straightforward way from more severe psychopathology or from an admixture of somatic and psychological symptoms. Nevertheless, we used only three questions on distress symptoms and one question on sick leave to recruit patients who had visited their PCP. We were convinced of the importance of undertaking this study, and for that reason might have underestimated the challenge of diagnosing SMDs. Thus, it is clear that the evidence base on criteria for the diagnosis of SMD in patients has to grow substantially.

Furthermore, the lack of effect might be due to the PCPs. They are the gatekeepers of health care and have extensive workloads. The MISS takes into account the time constraints under which a PCP works, as well as the position of a PCP as a generalist who does not have the capacity to apply highly specialised interventions. Nevertheless, the PCPs may have been too busy to carry out any intervention at all. Alternatively, the intervention might have been too minimal, or the training hours too short for the PCPs to actually learn the necessary skills. Unfortunately, except for detection and labelling of symptoms, the extent to which the PCPs actually applied their skills was not addressed in this study.

Ancillary analyses showed there was an interaction between the intervention and detection of SMDs, other mental health problems, or somatic problems. Both SMDs and other mental health problems were more frequently diagnosed by the PCPs in the MISS group than by the PCPs in the UC group. Since, as a result of the training, the MISS PCPs were more sensitive to mental disorders and more keen to diagnose SMDs in particular, it seems logical to expect that this result would be biased. It seems likely that the SMD patients in the MISS group were significantly less severely affected than the SMD patients in the UC group. This in turn could have caused differences in outcome of the SMD patients in the study group, giving a false (confounded) impression of the effect of the MISS. However, the SMD patients in the MISS group actually turned out to have higher baseline levels of symptoms than the SMD patients in the UC group (see Table S1). We tested baseline variables for confounding and as a result controlled for severity of symptoms, but that did not make the effect of the intervention in the SMD patients disappear. Nevertheless, it should be noted that this was an ancillary analysis, and because of the selection of the SMD patients by the PCPs' diagnosis, although it was a preplanned subgroup analysis, in no case can this result be regarded as evidence of a difference between the MISS group and UC group.

### Generalisability

We carried out a randomised controlled trial with only a few exclusion criteria and thereby allowed considerable variation due to context, diagnosis, and treatment, so flattering performances or overestimation of application are unlikely to be issues. Instead, this considerable variation increases the relevance of our results because it reflects routine clinical practice instead of ideal circumstances.

### Overall Evidence

As far as we know, this is the first randomised controlled trial to evaluate the effects of an intervention in primary care for SMDs with sick leave as a primary outcome. At the start of our study, the only comparable study had been performed in occupational health care by Jac van der Klink et al. [24], who reported high return-to-work rates after three months (78% in the intervention group and 63% in the UC group, *p* = 0.02). In our study, these rates were lower (51% in the MISS group and 57% in the UC group, *p* = 0.239). In the meantime, the results of one other randomised controlled trial performed in primary care have been published [1]. In that trial, social workers were trained to apply the intervention, while PCPs provided UC for patients in the other group. The study found no differences between the conditions. Return-to-work rates after three months were 37% in the intervention group and 40% in the UC group. The more favourable outcomes of this study seem to indicate that the occupational setting may be exceptional, and that the results may not equally apply to primary care. Moreover, our trial addressed a wider range of patients than the trial of van der Klink et al., who excluded patients with major depression or anxiety. The more beneficial effects of the MISS among patients with a PCP's diagnosis of SMD may indicate that these patients more strongly resemble the participants in the occupational study.

### Conclusions

This project represents a further step in the development of an evidence-based intervention for the treatment of distressed patients on sick leave. We were unable to show an effect of the MISS on duration of sick leave. In subgroup analyses a possible direction for further research was identified: namely, whether patients diagnosed with SMDs may benefit from an effect of the MISS on duration of sick leave. We feel that emphasis on functional rehabilitation of the patient is important, because continuation of sick leave may lead to chronicity and deterioration of symptoms. Unfortunately, diagnosis of SMD in primary health care turns out to be less straightforward than we expected, and the evidence base on criteria for this diagnosis will need to grow substantially before definite conclusions can be drawn. Researchers should take into account the importance of a diagnostic work-up to differentiate between common mental health problems, because there is still a lack of generally accepted criteria to diagnose “uncomplicated SMD” as a level of psychopathology.

Furthermore, continuing research should focus on the potential beneficial effects of the MISS; we need to investigate which elements of the intervention might be useful and which elements should be adjusted to make the MISS effective.

## SUPPORTING INFORMATION

CONSORT Checklist Click here for additional data file. (51 KB DOC)

Trial Protocol (English) Effectiveness of a Minimal Intervention for Stress-related mental disorders with Sick leave (MISS); Study protocol of a cluster randomised controlled trial in general practice. (165 KB PDF)

Trial Protocol (Dutch) Click here for additional data file. (137 KB PDF)

Table S1 Baseline Scores on 4DSQ Symptoms, and Number of Visits to the PCP, Stratified into Subgroups (41 KB DOC)

## Abbreviations

4DSQ: Four-Dimensional Symptom Questionnaire
CI: confidence interval
HR: hazard ratio
MISS: Minimal Intervention for Stress-related mental disorders with Sick leave
PCP: primary care physician
SD: standard deviation
SMD: stress-related mental disorder
UC: usual care
