# Supplementary material for: A Cluster-Randomised Trial Evaluating an Intervention for Patients with Stress-Related Mental Disorders and Sick Leave in Primary Care
Source: PLoS Clin Trials. 2007 Jun 1;2(6):e26. doi: 10.1371/journal.pctr.0020026 (PMC1885369; doi:10.1371/journal.pctr.0020026)
Supplement: Trial Protocol (Dutch) [file pctr.0020026.sd003.doc]

|  | *NHG fonds alledaagse ziekten* | *Projectaanvraag* |
| --- | --- | --- |
|  | Organisatie  Aanvrager  Postadres  Postcode + plaats  Telefoon  Fax  E-mail | EMGO-Instituut, VU-medisch centrum, Amsterdam  Prof.dr. W.A.B. Stalman  Van der Boechorststraat 7  1081 BT Amsterdam  020-4448199  020-4448361  W.Stalman.EMGO@med.vu.nl |
| 1 | Titel | Onderzoek naar de effectiviteit van een Minimale Interventie Strategie voor Surmenage (MISS) door de huisarts; een pragmatische gerandomiseerde gecontroleerde trial. |
| 2 | Samenvatting | Huisartsen hebben een taak bij het opvangen van overspannen mensen. Uit onderzoek is echter bekend dat huisartsen hierbij op twee gebieden problemen ondervinden. In de eerste plaats hebben zij moeite met het onderkennen van depressieve en angstige reacties bij patiënten met overspanningsklachten. Als gevolg daarvan krijgen de betreffende patiënten pas laat of niet een gerichte behandeling. In de tweede plaats lopen overspannen mensen een risico op langdurige arbeidsongeschiktheid door hun behoefte aan ‘rust’ en hun neiging passief te blijven en de problemen op hun beloop te laten. Uit ziekteverzuim-registraties blijkt dat 20% van alle overspannen patiënten een heel jaar verzuimt en wordt aangemeld voor de WAO. Huisartsen gaan vaak mee in het verlangen van de patiënt om met rust gelaten te worden, en verhogen zo – onbedoeld – het risico op langdurige arbeidsongeschiktheid. De laatste jaren wordt het onder deskundigen steeds duidelijker dat overspannen mensen een activerende benadering nodig hebben. Dat wil zeggen dat zij moeten worden aangemoedigd hun problemen aan te pakken en daar eventueel hulp bij te zoeken als ze het niet alleen aankunnen. Uitgaande van dit principe hebben wij voor de huisarts een 3-consulteninterventie ontwikkeld voor overspannen patiënten die hun werk verzuimen. In die 3 consulten moet de huisarts patiënten met depressieve en angstige reacties signaleren en een gerichte behandeling geven, de patiënt goede informatie over overspanning geven, de patiënt motiveren actief met zijn/haar problemen aan de slag te gaan en contact op te nemen met de bedrijfsarts. Ten slotte moet de huisarts het beloop in de eerste 4 weken in de gaten houden en bij het uitblijven van verbetering, de patiënt verwijzen. Wij willen het effect van die aanpak onderzoeken in 40 huisartspaktijken. De helft van de huisartsen krijgt scholing in de nieuwe aanpak, terwijl de andere helft blijft doen wat ze anders ook deden. 415 overspannen patiënten worden d.m.v. een aangepaste vorm van screening geworven en een jaar vervolgd met interviews en vragenlijsten. |

| 3 | Geplande duur 48 maanden  *(in maanden)* | Totale bedrag € |
| --- | --- | --- |

| 4. | Voornaamste invalshoek | **X** diagnose  **X** therapie  - voorlichting  - etiologie  - kwaliteit van leven |
| --- | --- | --- |

| 5. | Projectgroep  projectleider  onderzoeker  overig | naam en titel  Dr. B. Terluin  Drs. I.M. Bakker  Dr. H.P.J. van Hout  Mw. dr. A.H. Blankenstein  Mw. dr. I. Bramsen  Prof.dr. W. van Mechelen  Drs. J.R. Anema  Mw. Dr. P. van Oppen  Drs. J.J.L. van der Klink  Prof.dr. W.A.B. Stalman | discipline  huisarts  psycholoog  gezondheidsweten-schapper  huisarts  psycholoog  bedrijfsarts  bedrijfsarts  psycholoog/ gedragstherapeut  bedrijfsarts  huisarts | aanstelling bij  Huisartsgenees-kunde, VUmc  idem  Huisartsgenees-kunde, VUmc  idem  Med. Psychologie, VUmc  Soc. Geneeskunde, VUmc  idem/TNO-Arbeid  Psychiatrie, VUmc  NSOH, Utrecht  Huisartsgenees-kunde, VUmc | uren/wk  2  36  2  0,5  0,5  0,5  0,5  0,5  0,5  2 |
| --- | --- | --- | --- | --- | --- |

| 6 | Samenwerking anderen | Binnen het VU-medisch centrum: Sociale Geneeskunde (Bedrijfsgezondheidszorg), Psychiatrie, Medische Psychologie;  Buiten het VUmc: National School of Occupational Health (NSOH), Utrecht; TNO-Arbeid, Hoofddorp. |
| --- | --- | --- |
| 7. | relevante publicaties  *max. 10* | Terluin B, Van der Klink JJL. Surmenage. In: Van der Klink JJL (Red). Psychische problemen en de werksituatie. Amsterdam: NIA, 1995.  Terluin B. De Vierdimensionale Klachtenlijst (4DKL). Een vragenlijst voor het meten van distress, depressie, angst en somatisatie. Huisarts Wet 1996; 39:538-47.  Terluin B, Hekkelman-de Bie SC. Counseling bij ziekteverzuim door overspanning. Eerstelijns hulp bij disfunctioneren in het werk door psychische problemen. Maandblad Geestelijke Volksgezondheid 2000; 55: 1006-22.  Terluin B. Overspanning: rusten of juist aan de slag? [Commentaar] Huisarts Wet 2002; 45: 112-3.  Anema H, Buijs P, Van Amstel R, Van Putten D. Leidraad voor huisarts en bedrijfsarts bij de sociaal-medische begeleiding van arbeidsverzuim. Hoofddorp: TNO Arbeid, 2002.  Blankenstein AH. Somatising patients in general practice. Reattribution, a promising approach. Proefschrift, VU Amsterdam, 2001.  Bramsen I, Bleiker EMA, AHM Triemstra, et al. A Dutch adaptation of the Ways of Coping Questionnaire: factor structure and psychometric properties. Anxiety, Stress, and Coping 1995; 8: 337-52.  Stalman W, Van Essen GA, Van der Graaf Y, De Melker RA. The end of antibiotic treatment in adults with sinusitis-like complaints in general practice? A placebo-controlled double-blind randomized doxycycline trial. Br J Gen Pract 1997;47:794-9.  Van Hout H, Vernooij-Dassen M, Bakker K, et al. General practitioners on dementia: tasks, practices and obstacles. Patient Education and Counseling 2000; 39: 219-25.  Van der Klink JJL (red). Handelen van de bedrijfsarts bij werknemers met psychische klachten. Richtlijnen voor bedrijfsartsen. Eindhoven: NVAB, 2000. |

| 8. | Achtergrond  *Geef een heldere omschrijving van het probleem bij voorkeur gebaseerd op literatuur; geef de relevantie aan en formuleer een concrete vraagstelling* | Bij surmenage (overspanning) ondervinden huisartsen zowel een diagnostisch als een therapeutisch probleem.  *Diagnostisch* *probleem*. Huisartsen spreken van ‘surmenage’ (overspanning) bij patiënten die in hun ogen psychisch overbelast zijn door een te grote ‘draaglast’ in verhouding tot hun ‘draagkracht’, oftewel door te veel *stress*.(1) Het gaat daarbij vooral om overbelasting in het werk of de privé-situatie, en om levensgebeurtenissen en problemen. De overbelasting uit zich in typische ‘distress’-klachten (o.a. moeheid, gespannenheid, prikkelbaarheid en piekeren) en sociaal disfunctioneren, met name in ziekteverzuim.(2) Ongeveerde de helft van alle huisartspatiënten met een kenmerkend distresssyndroom wordt door de huisarts niet herkend.(3) Dit heeft vermoedelijk te maken met de (somatische) presentatie van patiënten en de vraaggerichte werkwijze van de huisarts. Bij patiënten die van de huisarts de diagnose surmenage krijgen, wordt in een op de vijf gevallen het klinisch beeld gecompliceerd door een meer of minder sterke depressieve en/of angstige reactie die de prognose negatief beïnvloedt, maar door de huisarts niet wordt herkend.(4)  *Therapeutisch probleem*. Surmenagepatiënten ‘kunnen het niet meer aan’ en willen vooral rust.(5) Het grote gevaar van rust is echter dat de patiënt de problematiek ‘oplost’ door **vermijding** ervan.(6) Dit lijkt de belangrijkste factor bij het ontstaan van langdurig ziekteverzuim en WAO (het risico op WAO is 20%(7;8)). Huisartsen rekenen het opvangen van surmenagepatiënten tot hun taak en besteden daaraan per patiënt gemiddeld bijna 2 consulten in 1-3 maanden tijd.(2;9) De huisarts beperkt zich vaak tot het voorschrijven van rust,(2) en riskeert daarmee mogelijke vermijdingstendensen bij de patiënt te versterken. Bij uiteenlopende problemen verergert vermijding als ‘copingstrategie’ de situatie.(10) Er bestaat een brede consensus voor een activerende, ondersteunende, oplossingsgerichte begeleiding van surmenage.(11;12) Een dergelijke benadering door bedrijfsartsen blijkt het ziekteverzuim met 30% te kunnen reduceren (Van der Klink, persoonlijke mededeling, 2001). De huisarts heeft als vertrouwenspersoon een uitstekende gelegenheid de patiënt ‘op het goede spoor’ te zetten,(13) resulterend in minder dan 1% WAO-instroom.(14;15)  *Mogelijke oplossing*. Op basis van de literatuur en praktijkervaring stellen wij een **Minimale Interventie Strategie voor Surmenage (MISS)** voor.(16-18) Uitgangspunt van de MISS is dat de huisarts met een minimale tijdsinvestering (3 consulten) een maximaal nuttig effect bereikt. De uitkomst van de patiënt is het resultaat van diverse factoren, w.o. de oorzaken van de problematiek, de reactiewijze van de patiënt en de interventies van de bedrijfsarts. In dit krachtenspel kunnen interventies van de huisarts een belangrijke invloed hebben vanwege zijn/haar vertrouwenspositie en het feit dat de meeste surmenagepatiënten hun huisarts in het begin van hun ziekteverzuim bezoeken. De MISS dient twee doelen: 1) het onderkennen van surmenage en het herkennen van depressieve en/of angstige reacties die gerichte interventies behoeven, en 2) het activeren en stimuleren van de patiënt om te voorkómen dat de problematiek op z’n beloop wordt gelaten. De MISS sluit goed aan bij recente ontwikkelingen op het gebied van psychische arbeidsongeschiktheid en arbocuratieve samenwerking.(19)  De **vraagstellingen** luiden:  1) Hebben patiënten met surmenageverschijnselen die hun werk verzuimen, een betere uitkomst indien hun huisarts is geschoold in de MISS dan wanneer hun huisarts de gebruikelijke zorg verleent?  2) Wat is de uitkomst na een jaar van patiënten met surmenageverschijnselen die hun werk verzuimen, en welke determinanten bepalen die uitkomst?  3) Gaan huisartsen die geschoold zijn in de MISS surmenageverschijnselen bij hun patiënten beter herkennen?  4) Gaan huisartsen die geschoold zijn in de MISS depressieve en angstige reacties bij patiënten met surmenageverschijnselen beter herkennen? |
| --- | --- | --- |

| 9 | Gebruikte literatuur | 1 Terluin B, Gill K, Winnubst J. Hoe zien huisartsen surmenage? Huisarts Wet 1992; 35:311-5.  2 Terluin B. Overspanning onderbouwd. Een onderzoek naar de diagnose surmenage in de huisartspraktijk. [Proefschrift] Utrecht: DETAM, 1994.  3 Terluin B. Naar een nieuwe indeling van psychosociale problemen in de eerste lijn. Een secundaire analyse van de symptomatologie van 396 huisartspatiënten en 67 cliënten van het algemeen maatschappelijk werk. Huisarts Wet 1998; 41:219-28,245.  4 Terluin B. Welke syndromen schuilen achter de diagnose surmenage? Een clusteranalyse. Huisarts Wet 1996; 39:358-65.  5 Terluin B, Troost J. Overspannen. Het verhaal van de patiënten. Medisch Contact 1993; 48:1544-6.  6 Terluin B. Overspanning: rusten of juist aan de slag? Huisarts Wet 2002; 45:112-3.  7 Schröer CAP. Verzuim wegens overspanning. Een onderzoek naar de aard van overspanning, de hulpverlening en het verzuimbeloop [Dissertatie]. Maastricht: Universitaire Pers, 1993.  8 Van Engers RW. Overspannen in de ziektewet; een onderzoek naar de oorzaken en het verloop van ziekteverzuim wegens overspanning. Amsterdam: TICA, 1995.  9 Terluin B, De Bakker DH, Verhaak P. Het gebruik van de diagnosen surmenage en depressie in de huisartspraktijk. Huisarts Wet 1993; 36:314-21.  10 Folkman S, Lazarus RS. Manual for the Ways of Coping Questionnaire. Palo Alto, CA: Consulting Psychologists Press, 1988.  11 Leidraad aanpak verzuim om psychische redenen. Commissie Psychische Arbeidsongeschikt-heid, 2001.  12 Bollen SH, Van der Klink JJL, Terluin B, Wijers JH. Snel weer aan het werk. De rol van artsen bij de aanpak van verzuim om psychische redenen. Medisch Contact 2002; 57:420-2.  13 Terluin B, Van der Klink JJL. Surmenage. In: Van der Klink JJL (Red). Psychische problemen en de werksituatie. Handboek voor een actieve sociaal-medische begeleiding. Tweede druk. Amsterdam: Nederlands Instituut voor Arbeidsomstandigheden NIA, 1995.  14 Terluin B. Surmenage in een huisartspraktijk. Een explorerend onderzoek. Huisarts Wet 1986; 29:261-4.  15 Terluin B. Surmenage in een huisartspraktijk. Over de wanverhouding tussen psychische belasting en belastbaarheid. Medisch Contact 1988; 43:1495-8.  16 Schippers GM, De Jong J. Motiverende gespreksvoering. MGv 2002; 57:250-65.  17 Gath D, Mynors­Wallis L. Problem­solving treatment in primary care. In: Clark DM, Fairburn CG, editors. Science and practice of cognitive behaviour therapy. Oxford: Oxford University Press, 1997.  18 Dryden W, Feltman C. Kortdurende hulpverlening. Baarn: Intro, 1996.  19 Anema H, Buijs P, Van Amstel R, Van Putten D. Leidraad voor huisarts en bedrijfsarts bij de sociaal-medische begeleiding van arbeidsverzuim. Hoofddorp: TNO Arbeid, 2002.  20 Van der Klink JJL. Handelen van de bedrijfsarts bij werknemers met psychische klachten. Richtlijnen voor bedrijfsartsen. Eindhoven: NVAB, 2000.  21 Terluin B. De Vierdimensionale Klachtenlijst (4DKL). Een vragenlijst voor het meten van distress, depressie, angst en somatisatie. Huisarts Wet 1996; 39:538-47.  22 Van der Zee K, Sanderman R, Heyink J. De psychometrische kwaliteiten van de MOS 36-item Short Form Health Survey (SF-36) in een Nederlandse populatie. T Soc Gezondheidsz 1993; 71:183-91.  23 Bramsen I, Bleiker EMA, Triemstra AHM, Van Rossum MG, Van der Ploeg HM. A Dutch adaptation of the Ways of Coping Questionnaire: factor structure and psychometric properties. Anxiety, Stress, and Coping 1995; 8:337-52. |
| --- | --- | --- |
| 10 | Methoden  *Design*  *Gedetailleerde omschrijving van onderzoekseenheid (patiënt/huisarts), wijze van selectie, meetinstrumenten, uitkomstmaten, confounding.*  *Bij interventie onderzoek ook beschrijving interventie.*  *Vervolg methoden*  *Analyse*  *wordt er van een bestaande infrastructuur gebruik gemaakt? Zo ja, zijn daar al definitieve toezeggingen gedaan* | **Design**: Pragmatische gerandomiseerde gecontroleerde trial. De interventie is gericht op de huisarts; deze krijgt een training in de best mogelijke aanpak van surmenage in de beperkte tijd van 3 consulten. Het effect van de interventie wordt afgemeten aan de uitkomst van patiënten met surmenageverschijnselen die hun werk verzuimen.  **Onderzoekspopulatie**. De patiënten voor het onderzoek worden als volgt geworven:  - Aan de hand van uitdraaien van spreekuren worden patiënten geselecteerd voor een schriftelijke screening. Uitgesloten worden patiënten die de Nederlandse taal niet beheersen, en patiënten die ernstig suïcidaal, manisch, psychotisch of terminaal ziek zijn.  - De patiënten ontvangen vanuit de praktijk namens de huisarts een korte enquête per post met vragen over distressklachten, betaald werk en ziektverzuim.  - Op basis van deze screening worden patiënten geselecteerd die voldoen aan de volgende criteria: distressklachten op surmenageniveau, betaald werk, ziekteverzuim korter dan 3 maanden, leeftijd 20-60 jaar.  - De geselecteerde patiënten worden telefonisch benaderd met informatie over het onderzoek; schriftelijke informatie wordt per post toegezonden. Nadat de patiënt informed consent heeft gegeven, wordt een afspraak voor een telefonisch baseline interview gemaakt.  De argumenten voor deze selectieprocedure waarbij een relatief heterogene patiëntengroep grotendeels buiten de huisarts om wordt geworven, zijn: 1) huisartsen hebben moeite met het onderscheiden van relevante subgroepen;(3) 2) er bestaan veel mengbeelden van distress, depressie en angst;(3) 3) dit is de beste manier om in de interventie- en controlegroepen vergelijkbare patiënten te krijgen.  **Randomisatie** vindt plaats op huisartspraktijk-niveau. De interventiehuisartsen (n=20) worden getraind in de MISS. De controlehuisartsen (n=20) krijgen alleen informatie over het onderzoek gedurende een bijeenkomst van circa 1,5 uur.  **Interventie**: De interventiehuisartsen worden getraind in de vaardigheden die nodig zijn voor het succesvol uitvoeren van de MISS (de training bestaat uit 2 dagdelen en 2 opfrisbijeenkomsten van 2 uur). Het MISS-protocol behelst de volgende onderdelen:  *1^e^ consult*: a) Signalering en diagnostiek van surmenageverschijnselen. b) Signalering en diagnostiek van depressieve en angstige reacties. c) Uitleg over de diagnose. d) Advisering t.a.v. het in kaart brengen en (later) aanpakken van de stressfactoren (al of niet met hulp van derden), naast rust en afleiding. e) Gerichte behandeling van depressieve en angstige reacties d.m.v. medicamenteuze therapie of verwijzing. De uitleg en advisering door de huisarts wordt ondersteund door praktische schriftelijke informatie op maat, afhankelijk van de gesignaleerde problematiek.  *2^e^ consult (na 1 week)*: a) Beoordeling of de patiënt de informatie en adviezen begrepen heeft en op het goede spoor zit. b) Informatie over de functie van de bedrijfsarts bij psychische klachten.(20) c) Advies om contact op te nemen met de bedrijfsarts conform de Leidraad voor huisarts en bedrijfsarts.(19)  *3^e^ consult (na 4 weken)*: Evaluatie van het beloop. Als zich binnen 4 weken geen verbetering aftekent, bespreekt de huisarts verwijzing naar een psychosociale hulpverlener – liefst in overleg met de bedrijfsarts conform de Leidraad voor huisarts en bedrijfsarts.(19)  **Controleconditie**: Care as usual.  **Uitkomstmaten:** *primair*: duur ziekteverzuim; *secundair*: klachten (4DKL),(21) sociaal functioneren (SF-36),(22) WAO-aanmelding, verlies van werk, medische consumptie.  **Metingen**: *Patiënten*: Baseline interview: persoonsgegevens, medische gegevens, psychosociale problemen en levensgebeurtenissen,(2) coping,(23) 4DKL-profiel,(21) SF-36.(22) Follow-up na 6 weken, 3, 6 en 12 maanden: telefonische interviews: ziekteverzuim, WAO-aanmelding, verlies van werk, contacten met HA en BA, medische consumptie; vragenlijsten per post: 4DKL, SF-36, coping.  *Huisarts*: Dossieronderzoek na 3 en 12 maanden:diagnosen, verwijzingen, contacten met BA, (MISS-)acties HA.  *Bedrijfsarts:* Telefonisch interview: acties BA, contacten met HA.  **Analyse:** Intention to treat, multilevel analyse, survival analyse.  **Haalbaarheid:** Een huisarts heeft 100 consulten met volwassenen per week. 32 patiënten hebben een psychosociaal probleem met distressklachten.(3) Daarvan komen 8 patiënten in aanmerking voor het onderzoek. Bij 50% respons kan 1 huisartspraktijk 4 patiënten per week includeren.  *Powerberekening*: [Aan de powerberekening wordt nog gewerkt.] |

| 11 | Medisch ethische toetsing  *Is het project bij interventie onderzoek getoetst? Zo ja, door wie?*  *Indien getoetst, toestemming meesturen* | Het onderzoeksvoorstel wordt voorgelegd aan de Medisch-Ethische Commissie van de Vrije Universiteit Amsterdam |
| --- | --- | --- |

| 12 | Werkplan per maand | - maand 1-12: voorbereiding onderzoek / werving huisartsen. De onderzoeksgroep heeft contacten met een groep gemotiveerde huisartsen in Haarlem en Almere met volledig geautomatiseerde praktijken.  - maand 12-17: training huisartsen in 2 groepen  - maand 13-22: inclusie patiënten, baseline interviews  - maand 15-34: follow-up gegevens patiënten verzameling  - maand 15-34: gegevens huisartsen en bedrijfsartsen verzamelen  - maand 19-34: gegevens invoeren en schonen  - maand 25-48: analyse gegevens en publicaties |
| --- | --- | --- |

| 13 | Totale budget | Fonds Alledaagse Ziekten | Eigen bijdrage Instituut | Andere fondsen |
| --- | --- | --- | --- | --- |

| 17 | Geef samenvatting in het Engels (250 woorden) | General practitioners (GPs) experience two kinds of problems in dealing with distressed patients. Firstly, GPs find it difficult to recognize depressive and anxious reactions. Secondly, GPs often do not recognize the patients’ tendencies to avoid problems, thereby increasing the risk for prolonged disability. Recently, experts agree that distressed people should not be left alone for as long as it takes, but instead should be stimulated to take an active approach to their problems, and to seek professional assistance if they cannot cope with it on their own. Therefore, we have developed an intervention package for the GP to deal with distressed patients. Within the limits of three ten-minutes consultations, the GP should 1) detect significant depression and anxiety, to deal with it separately, 2) educate the patient about distress and the best ways to cope with the situation, 3) advice the patient to see his/her occupational physician, and 4) evaluate any progress four weeks later, and refer the patient to a psychological professional when progress is absent. This proposal contains a pragmatic randomized trial in which twenty randomly allocated GPs shall be trained in the intervention skills, whereas twenty other GPs are allocated to the control condition. A total of 415 distressed patients who are on sick leave are selected from the general practice attenders, by means of a screening procedure. The patients shall be followed up for one year. The outcome measures are duration of sick leave, application for disability benefit, unemployment, psychological symptoms, and social functioning. |
| --- | --- | --- |
| 18 | Geef in een pakkende zin het belang van het onderzoek weer t.b.v. rapportages aan anderen. | Het belang van dit onderzoek is om vast te stellen wat het nuttig effect is van het leren aan huisartsen van een gerichte, weinig tijd kostende, diagnostische en therapeutische pakket-interventie voor patiënten met overspanningsverschijnselen. Het nuttig effect wordt afgemeten aan de duur van het werkverzuim, de klachten en het sociaal functioneren van patiënten. |
