# Supplementary material for: A Cluster-Randomised Trial Evaluating an Intervention for Patients with Stress-Related Mental Disorders and Sick Leave in Primary Care
Source: PLoS Clin Trials. 2007 Jun 1;2(6):e26. doi: 10.1371/journal.pctr.0020026 (PMC1885369; doi:10.1371/journal.pctr.0020026)
Supplement: Table S1 — (41 KB DOC) [file pctr.0020026.st001.doc]

# Table S1. Baseline scores on 4DSQ symptoms, and number of visits to the PCP, stratified into subgroups

|  | **MISS** | **UC** |
| --- | --- | --- |
| **Subgroup SMD** |  |  |
| Distress | 20.56 | 19.08 |
| Depression | 3.94 | 2.64 |
| Anxiety | 6.31 | 4.50 |
| Somatization | 14.52 | 10.86 |
|  |  |  |
| Mean # of visits to the GP¹ | 2.60 | 2.34 |
| **Subgroup other mental health problems** |  |  |
| Distress | 17.47 | 20.41 |
| Depression | 2.89 | 3.93 |
| Anxiety | 4.69 | 6.41 |
| Somatization | 10.25 | 14.54 |
|  |  |  |
| Mean # of visits to the GP¹ | 3.13 | 3.20 |
| **Subgroup somatic diagnosis** |  |  |
| Distress | 17.80 | 17.03 |
| Depression | 2.80 | 2.74 |
| Anxiety | 6.12 | 3.84 |
| Somatization | 12.64 | 12.14 |
|  |  |  |
| Mean # of visits to the GP¹ | 2.03 | 1.88 |

¹ Counted from the day of sick leave + 3 months
